# Exploratory Ultrasound Analysis of the Diaphragm and Respiratory Capacity in Women with Primary Dysmenorrhea: A Cross-Sectional Observational Study

**DOI:** 10.3390/mps8010015

**Published:** 2025-02-04

**Authors:** Rebeca del Prado-Álvarez, María García-Arrabé, Ángel González-de-la-Flor, Marta de la Plaza San Frutos, Jaime Almazán-Polo, Cecilia Estrada-Barranco

**Affiliations:** Department of Physiotherapy, Faculty of Medicine, Health and Sport, European University of Madrid, 28670 Madrid, Spain; rebeca.delprado@universidadeuropea.es (R.d.P.-Á.); maria.gararrabe@universidadeuropea.es (M.G.-A.); marta.delaplaza@universidadeuropea.es (M.d.l.P.S.F.); jaime.almazan@universidadeuropea.es (J.A.-P.); cecilia.estrada@universidadeuropea.es (C.E.-B.)

**Keywords:** primary dysmenorrhea, diaphragmatic function, respiratory mechanics, ultrasound imaging, chronic pain, intercostal distance, spirometry

## Abstract

Primary dysmenorrhea (PD) is a common gynecological condition characterized by menstrual pain without underlying pelvic pathology. It has been linked to functional and structural changes in the core musculature, but limited evidence exists regarding its association with diaphragmatic and respiratory mechanics. This study aimed to elaborate on these potential associations by assessing the diaphragmatic structure and respiratory function in women with PD compared to healthy controls, utilizing ultrasound imaging, spirometry and respiratory pressure measurements. Methods: An observational, cross-sectional study was conducted with 44 female participants (22 with PD and 22 healthy controls). Diaphragmatic structure was evaluated through ultrasound, measuring the intercostal distance, diaphragmatic thickness, and diaphragmatic excursion at rest and during maximum voluntary contraction. Spirometric assessments included forced vital capacity (FVC), forced expiratory volume in the first second (FEV1), and the FVC/FEV1 ratio, along with measurements of maximum inspiratory pressure (MIP) and maximum expiratory pressure (MEP). Group differences were analyzed using Student’s *t*-test and effect sizes were reported with Cohen’s d. Results: No significant differences were observed between the groups in diaphragmatic thickness, diaphragmatic excursion, or global respiratory capacity (*p* > 0.05). However, women with PD presented a significant reduction in the left intercostal distance both at rest (*p* = 0.035, d = 0.56) and during contraction (*p* = 0.039, d = 0.54). No other significant group differences were detected. Conclusions: While primary dysmenorrhea does not appear to affect overall diaphragmatic function or respiratory capacity, it is associated with subtle localized changes in the left intercostal dynamics. These findings suggest a potential compensatory mechanical adaptation rather than global respiratory dysfunction. Further longitudinal studies with larger sample sizes are needed to explore the clinical significance of these findings.

## 1. Introduction

Primary dysmenorrhea (PD), also known as menstrual pain, is one of the most common gynecological conditions affecting women of reproductive age, with a global prevalence estimated to range from 45% to 95% [1], and it can be disabling in 7% to 15% of cases [2]. PD is diagnosed by exclusion, as it is characterized by symptoms that are not associated with any underlying pelvic pathology [3]. Its etiology is linked to an increased secretion of prostaglandins during the luteal phase of the menstrual cycle; this process, combined with a decrease in estrogen levels, can trigger dysfunctional uterine contractility [4] The pain reaches its peak intensity during the first two days of the menstrual cycle, coinciding with the shedding of the endometrial lining. The clinical pain pattern is diverse, ranging from a dull, constant ache to spasmodic, cramp-like sensations. Although the pain is primarily localized in the pelvic region, it often radiates to other areas, such as the lumbar region, groin, or the proximal third of the thighs [4,5]. In contrast, secondary dysmenorrhea is associated with underlying pelvic pathology, such as endometriosis or fibroids, and typically involves more severe or chronic pain [6].

Various functional and psychological alterations associated with lumbopelvic pain during the menstrual cycle have been observed in women with PD [5,7]. Functionally, some studies have reported that women with PD may experience changes in muscle activation patterns [8] and muscle endurance, which could affect their functional capacity [9,10]. Additionally, psychological factors such as stress and catastrophizing may exacerbate the pain perception in women with PD, thereby impacting their quality of life [5,7]

Moreover, anatomical changes in the core musculature have also been identified. The core is composed of the abdominal muscles, spinal erector muscles, pelvic floor muscles, and the thoracic diaphragm. This group of muscles functions as a corset, playing an essential role in body stability and postural control [11], as well as in genitourinary [12,13] and respiratory [14,15] functions.

A previous study [16] found significant differences in the thickness of certain abdominal muscles between women with PD and those without this condition. Specifically, the rectus abdominis (RA) and external oblique (EO) muscles showed significantly lower thickness values in the PD group compared to the control group, with statistical significance (*p* < 0.05). These findings suggest a possible relationship between PD and selective weakening of the superficial abdominal muscles, likely due to chronic pain and reduced physical activity, which may lead to muscle atrophy. This aligns with the findings of previous research that has highlighted structural muscle alterations, such as atrophy or fatty infiltration, in the presence of chronic pain.

While the influence of abdominal and pelvic floor muscles on pelvic pain has been extensively studied, as evidenced by the aforementioned research, there is a notable gap in the literature regarding the role of the diaphragm in women with PD. Evaluating the diaphragm through ultrasound and analyzing its relationship with pulmonary functional capacity could provide a more integrated perspective on the biomechanical alterations observed in patients with PD. This line of research is particularly relevant as it complements existing approaches by exploring the potential role of the diaphragm in regulating intra-abdominal pressure and its impact on functionality and pain. During inspiration, the diaphragm descends into the abdominal cavity. The contraction of the diaphragm triggers synergistic contractions of all the muscles within this functional unit, including the urogenital diaphragm, which acts as a container for intra-abdominal pressures [17,18]. Structural modifications of the thoracic diaphragm could influence the clinical symptoms experienced by women with dysmenorrhea, as well as produce functional alterations in pulmonary capacity.

Additionally, previous studies have demonstrated that the activation of the sympathetic nervous system in chronic pain situations can alter cardiorespiratory function, leading to changes in breathing patterns, inducing hyperventilation, and heightening the stress response, thereby perpetuating a vicious cycle of pain and physiological alterations [19,20,21,22].

Given this context, the objective of the present study is to assess the structure and functionality of the diaphragm in women with and without PD through ultrasound, as well as to analyze pulmonary capacity using spirometry tests and the assessment of inspiratory and expiratory pressures.

## 2. Methodology

### 2.1. Study Design

An observational, cross-sectional study was conducted to evaluate the ultrasonographic differences in diaphragmatic structure and respiratory function between women with primary dysmenorrhea (PD) and women without PD (control group).

The study was approved by the Ethics Committee of the European University of Madrid (UEM) under the code CIPI 23/146. All procedures conducted in the study followed the guidelines of the Declaration of Helsinki and its amendments. Participants were informed about the objectives and procedures of the study, and they provided written informed consent before participating.

### 2.2. Participants

The study included a total of 44 female participants, divided into two groups: 22 women with PD and 22 women without PD. The inclusion criteria for participants were as follows: being female, aged between 18 and 35 years, having a diagnosis of PD (confirmed by the medical service of the European University of Madrid), and not having any previous respiratory or musculoskeletal pathologies. The exclusion criteria included the use of contraceptive medications, medications affecting respiratory function, and the presence of any comorbidities.

### 2.3. Measurement Procedure

The measurement protocol was conducted in several consecutive stages to ensure precise and consistent data collection. First, participants underwent a basic assessment where their age was recorded, and their height, weight, and body mass index (BMI) were measured.

**Figure 1:**
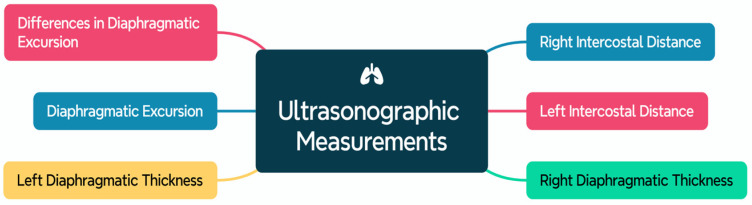
Ultrasound measurements.

Next, diaphragmatic ultrasound measurements were performed using a high-resolution portable ultrasound device (Figure 1). The measurements included intercostal distance in both resting and maximum voluntary contraction states on both the right and left sides. Additionally, diaphragmatic thickness was measured under the same conditions (rest and contraction), as well as diaphragmatic excursion, calculated as the difference between resting and contracted positions during deep breathing.

**Figure 2:**
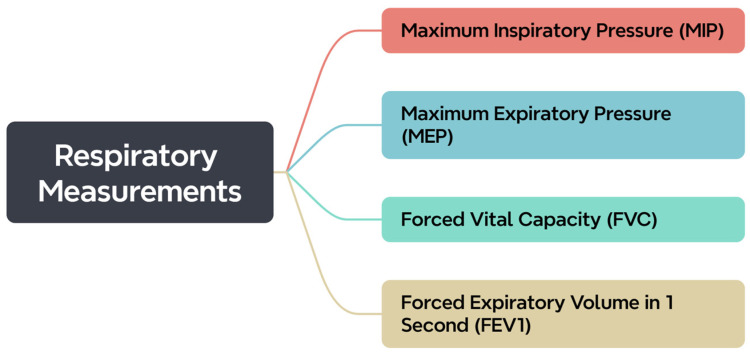
Respiratory measurements.

Finally, pulmonary capacity was assessed through spirometry and maximum respiratory pressure measurements (Figure 2). The variables analyzed included maximum inspiratory pressure (MIP), maximum expiratory pressure (MEP), forced vital capacity (FVC), forced expiratory volume in the first second (FEV1), and the FVC/FEV1 ratio as key indicators of pulmonary function.

### 2.4. Sonographic Measurements

Sonographic assessments were performed by an experienced physical therapist with more than 5 years of expertise in musculoskeletal ultrasound, who was blinded to participants’ group allocation (J.A.P.), using high-quality ultrasound equipment (Logiq S7 Expert, GE Healthcare, Chicago, IL, USA). Two types of probes were utilized: a broad-spectrum linear matrix array probe (ML6-15 H40452LY, GE Healthcare, Chicago, IL, USA) 50 mm field of view) with a frequency range of 5–15 MHz for transcostal evaluation and diaphragm thickness measurements, and a broad-spectrum convex probe (C1-5-D H40432LE, 70° field of view, GE Healthcare, Chicago, IL, USA) with a frequency range of 1–6 MHz for transhepatic evaluation and diaphragmatic excursion measurements. The linear probe was used to assess diaphragm thickness via the intercostal spaces. For the transcostal examination, a preset configuration was applied with a depth of 3 cm, a frequency of 12 MHz, and a single focus at a depth of 2 cm [23]. Diaphragmatic excursion during transhepatic examination was evaluated using a preset of 23 cm in depth, a frequency of 4 MHz, and one focus located at a depth of 21 cm [24].

Participants were instructed to remain in a relaxed supine position during the ultrasound scan. Both maximum relaxed inspiration (T.^insp.^) and maximum relaxed expiration (T.^exp.^) were assessed for each hemi-diaphragm during transcostal assessment for diaphragm thickness and intercostal space evaluation, and only for the right hemi-diaphragm during transhepatic maneuver, in order to calculate the difference between both breathing states (T.^dif.^ = T.^insp.^ − T.^exp.^). Both the transcostal and transhepatic evaluation methods demonstrated excellent reliability. The ICC values ranged from 0.939 to 0.971, with SEM values spanning from 0.023 cm to 0.725 cm and MDC values from 0.064 cm to 2.009 cm, confirming the reproducibility and clinical utility of these ultrasound imaging approaches [23,24,25].

**Figure 3:**
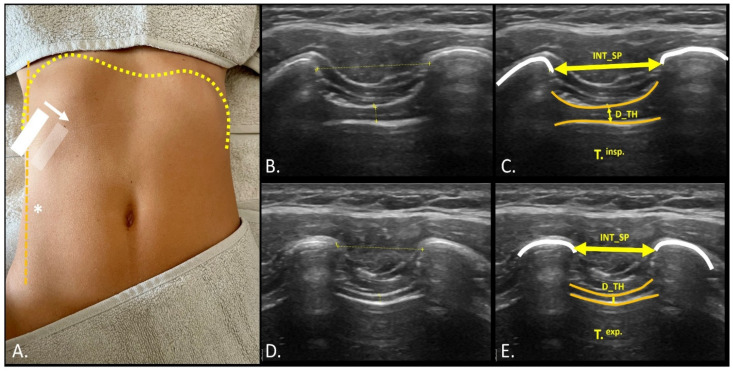
Evaluation of changes in diaphragmatic thickness and intercostal distance using the transcostal maneuver: (**A**) Using the midaxillary line (*) as a reference, the probe was positioned perpendicularly to the 11th–12th intercostal spaces to assess diaphragm thickening during breathing. In cases where the costodiaphragmatic space encroached and obstructed diaphragm visualization, the probe was adjusted forward (transparent box and white arrow). The evaluation was conducted at the maximum intercostal distance and diaphragmatic thickness achieved during maximum inspiration (**B**,**C**; T.^insp.^) and maximum expiration (**D**,**E**; T.^exp.^) in basal breathing. Abbreviations: DP_TH, diaphragmatic thickening; INT_SP, intercostal space; T.^insp.^, inspiratory time during basal breathing; T.^exp.^, expiratory time during basal breathing.

**Figure 4:**
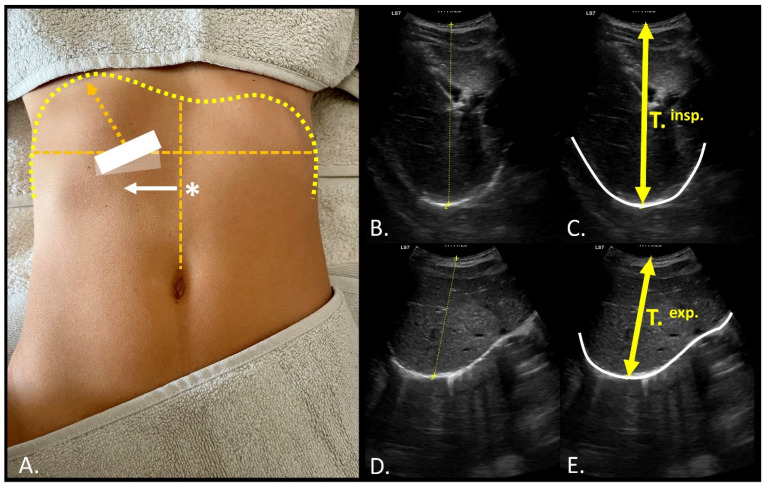
Assessment of diaphragmatic excursion using the transhepatic maneuver; (**A**) Using the umbilical line (*) as a reference, the probe is moved laterally to project directly onto the right diaphragmatic dome at the level of the 9th–10th ribs (white arrow). The probe is positioned at a tilt angle of 30–40° on the sagittal plane, with efforts to maintain the ultrasound beam projection as vertical as possible during respiratory phases. Pivot movements of the probe are adjusted to optimize visualization during the expiratory (**B**,**C**) and inspiratory (**D**,**E**) phases, while holding the probe firmly with both hands to minimize displacement. Abbreviations: T.^insp.^, inspiratory time during basal breathing; T.^exp.^, expiratory time during basal breathing.

To measure diaphragm thickness, the probe was positioned perpendicular to the intercostal space between the 10th and 11th ribs, at the level of the midaxillary line. If visibility of the diaphragm was compromised due to entry into the costodiaphragmatic angle during inhalation, the probe was repositioned distally along the intercostal space to visualize the most anterior portion of the diaphragmatic insertions (Figure 3A, transparent box and white arrow) [25]. The assessment of the intercostal distance, both in inspiration (T.^insp.^) and expiration (T.^exp.^), was carried out at the point of greatest intercostal distance from the last visible edge of the cortex of the 11th rib bone and from the last visible point of the cortex of the 12th rib (Figure 3). Both distances were used as references for diaphragm thickness measurements at the center of the same distance. Right hemi-diaphragm excursion was assessed using a transhepatic approach by placing the convex probe beneath the right costal margin along the mid-clavicular line, with the ultrasound beam directed towards the mid-posterior aspect of the diaphragm. A consistent angle from 40° to 30° was maintained to evaluate the right dome diaphragmatic excursion, ensuring that measurements were taken at the same anatomical reference point (Figure 4) [25]. Three images were captured at T.^insp.^ and T.^exp.^, and diaphragmatic excursion was calculated as the distance between the remotest diaphragmatic point (dome) and the midpoint of the convex probe. Video sequences were recorded for subsequent analysis using the open-source software ImageJ—Fiji (U.S. National Institutes of Health, Bethesda, MD, USA), enabling offline measurement of diaphragm thickness and excursion [26].

### 2.5. Pulmonary Function Measurements

Pulmonary function was assessed using spirometry (Easyone^®^ Connect, ndd medical technologies, Andover, MA, USA). Spirometric variables included forced vital capacity (FVC), forced expiratory volume in the first second (FEV1), and the FEV1/FVC ratio. The spirometry test was conducted following standard guidelines [27] to ensure the reproducibility and reliability of the measurements. At least three valid measurements of each parameter were obtained, and the best of the maneuvers was selected for analysis.

Maximum inspiratory and expiratory pressures (MIP and MEP) were measured using a pressure manometer (Micro RPM^®^, MicroMedical-Carefusion, Rochester, UK). The values were recorded after three consecutive attempts, in accordance with international standards [28].

### 2.6. Statistical Analysis

Statistical analyses were conducted using SPSS software version 29 for Windows. the normality of the data distribution was assessed with the Shapiro–Wilk test and by examining histograms. The Levene test was used to assess the homogeneity of variances. A *p*-value below 0.05 was interpreted as indicating a non-normal distribution, while a *p*-value above 0.05 indicated a normal distribution. Descriptive statistics were then performed to characterize the sample, using the mean and standard deviation for normally distributed variables and median and interquartile range for variables that were not normally distributed. Differences between the control and dysmenorrhea groups were analyzed using Student’s *t*-test for independent samples for ultrasound and respiratory measurements. Cohen’s d was used to measure the effect size, with values interpreted as small (0.2), medium (0.5), and large (0.8). Statistical significance was set at *p* < 0.05.

## 3. Results

The study included a total of 44 female participants, with 22 in the dysmenorrhea group (DG) and 22 in the control group (CG). Overall, the sample had an average age of 26.16 ± 6.97 years, an average weight of 61.24 ± 9.88 kg, an average height of 164.91 ± 6.46 cm, and an average BMI of 22.44 ± 2.97 kg/m^2^. In comparison, the DG had an average age of 27.18 ± 6.51 years and in the CG this was 25.14 ± 7.41 years. The average weight for the DG was 61.32 ± 9.02 kg versus 61.16 ± 10.89 kg for the CG. The average height was 166.00 ± 5.98 cm for the DG and 163.82 ± 6.86 cm for the CG. The mean BMI was 22.18 ± 2.64 kg/m^2^ for the DG and 22.70 ± 3.31 kg/m^2^ for the CG. Statistical analysis revealed no significant differences between the groups for age (*p* = 0.337), weight (*p* = 0.958), height (*p* = 0.267), or BMI (*p* = 0.567).

**Figure 5:**
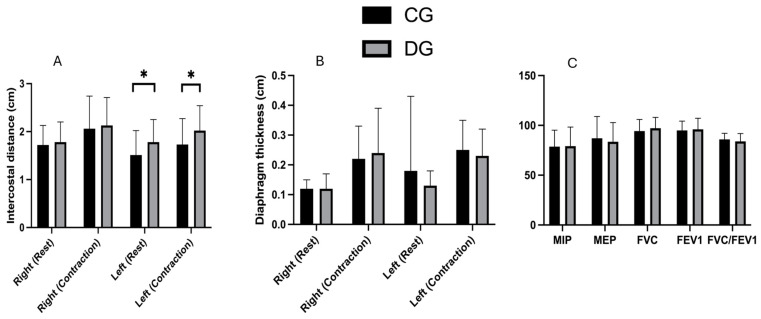
Comparison of intercostal distance (**A**), diaphragm thickness (**B**), and respiratory parameters (**C**) between groups. Abbreviations: CG, control group; DG, dysmenorrhea group; * *p*-value < 0.05.

Table 1 and Figure 5A,B show the data on the ultrasound differences observed between the CG and the DG. Left intercostal distance at rest showed a significant difference (*p* = 0.035), with a moderate effect size and a mean difference of −0.27. Left intercostal distance at contraction demonstrated a significant difference (*p* = 0.039), with a moderate effect size and a mean difference of −0.29. No significant differences were found for the rest of the variables (*p* > 0.05).

Table 2 and Figure 5C show the respiratory measurements for the total sample and the differences between groups, with no significant differences observed in any of the values.

In the DG, a comparison was performed based on pain intensity (moderate pain [Visual analog scale (VAS) = 5–7] and severe pain [VAS = 8–10]); no statistically significant differences were found in any of the variables studied.

## 4. Discussion

Our study provides relevant data on the relationship between PD and diaphragmatic and respiratory function in women, highlighting that no significant differences were observed in diaphragmatic movement or overall respiratory function when comparing women with PD and healthy controls. However, we identified a significant difference in the left intercostal distance both at rest (*p* = 0.035) and during contraction (*p* = 0.039), suggesting a localized alteration in intercostal mechanics.

These findings contrast with previous studies that have associated chronic pain with postural alterations, inefficient breathing patterns, and diaphragmatic dysfunction [29,30]. In this case, although changes in intercostal dynamics were observed, global respiratory capacity and diaphragmatic movement remained unaffected. This could indicate that PD does not substantially compromise overall respiratory function but induces specific mechanical adaptations that may represent localized responses to pain.

The alteration in the left intercostal distance could be related to a compensatory change in respiratory mechanics due to chronic pelvic pain, as suggested by previous studies in other chronic pain conditions [31]. These changes might represent adaptive strategies to minimize pain during deep breathing or to optimize mobility in other regions [32,33]. Additionally, the literature has noted that respiratory mechanical changes, such as shallow breathing, may lead to intercostal muscle dysfunction, contributing to muscle tension and chronic pain [34,35].

Our findings also align with those reported by Dogru et al. [36], who did not identify respiratory function impairments in women with PD. However, our study adds a more detailed analysis by including specific measurements of intercostal dynamics, revealing subtle differences that could be overlooked in assessments limited to spirometric evaluations.

From a clinical perspective, the observed difference in the left intercostal distance may be relevant to understanding how women with PD adapt their respiratory mechanics. Although these adaptations appear localized and do not imply global respiratory dysfunction, they might influence posture or the ability to perform deep breathing effectively. Therefore, while direct intervention on the diaphragmatic musculature may not be justified, physical therapists should consider these changes when planning management strategies, prioritizing pain reduction and improving overall quality of life. To enhance reproducibility and ensure that these findings can guide future interventions, standardized descriptions of imaging and measurement procedures, as well as consistent methodological protocols, are crucial.

### 4.1. Strengths and Limitations

The use of ultrasound as a non-invasive, real-time tool is a strength of our study, as it allows for precise evaluation of respiratory and diaphragmatic mechanics while minimizing participant discomfort [37]. Additionally, the inclusion of both static and dynamic measurements of the diaphragm and intercostal structures provides a comprehensive assessment of respiratory function beyond standard spirometric evaluations. The rigorous methodology, including the use of validated tools for respiratory assessment and standardized data collection protocols, further enhances the reliability of the results. Moreover, the recruitment of a well-defined sample of women with PD, carefully matched with healthy controls, contributes to the internal validity of the findings and reduces potential confounding factors.

Although this study addresses a relatively underexplored aspect of PD, expanding current knowledge on the potential mechanical adaptations associated with chronic pelvic pain conditions, it presents some limitations that should be acknowledged. Firstly, the cross-sectional design restricts the ability to establish causal relationships between PD and the observed changes in intercostal mechanics. Longitudinal studies are necessary to clarify whether these mechanical alterations are a consequence of PD or represent pre-existing anatomical or functional variations. Secondly, the relatively small sample size may have limited the statistical power to detect more subtle differences in diaphragmatic and respiratory function. Expanding the sample size in future studies would improve the generalizability of the findings. Thirdly, while ultrasound imaging was a reliable, non-invasive tool for assessing diaphragmatic and intercostal mechanics, its operator-dependent nature requires specialized training and standardized protocols to minimize variability in future research. Finally, this study did not explicitly control for potential confounding factors, such as exercise and dietary habits, which could influence ventilatory mechanics.

### 4.2. Future Directions

Further investigations should explore the long-term impact of PD on respiratory mechanics and whether the localized intercostal alterations observed in this study influence functional outcomes such as posture, breathing patterns, and physical performance. Additionally, examining the effectiveness of specific therapeutic interventions, such as breathing retraining or core stability programs, could offer insights into whether targeted physiotherapy strategies may help optimize musculoskeletal function and pain management in women with PD. Furthermore, future studies should examine the impact of the time elapsed since menarche and the duration of menstrual experience on dysmenorrhea symptoms. In addition, investigating how pain intensity affects the severity and management of primary dysmenorrhea could provide valuable insights into the factors influencing the clinical presentation and treatment of the condition.

## 5. Conclusions

This study suggests that PD is not associated with significant alterations in diaphragmatic function or overall respiratory capacity, although localized changes in the left intercostal distance were identified. These findings support the idea that PD does not generate a widespread impact on respiratory function but emphasize the need for further research to explore the clinical relevance and underlying mechanisms of these specific intercostal alterations.

## Figures and Tables

**Table 1:** Ultrasound measurements of the total sample and differences between groups.

Ultrasound Measurements		Total Sample (n = 44)	CG (n = 22)	DG (n = 22)	*p*-Value	Cohen’s d	Mean Difference (95% CI)
Right Intercostal Distance	Rest	1.75 ± 0.42	1.72 ± 0.41	1.78 ± 0.42	0.300	0.16	−0.07 (−0.32, 0.19)
	Contraction	2.09 ± 0.63	2.06 ± 0.68	2.13 ± 0.58	0.369	0.10	−0.06 (−0.45, 0.32)
Left Intercostal Distance	Rest	1.65 ± 0.49	1.51 ± 0.51	1.78 ± 0.47	**0.035**	0.56	−0.27 (−0.57, 0.02)
	Contraction	1.87 ± 0.53	1.73 ± 0.54	2.02 ± 0.52	**0.039**	0.54	−0.29 (−0.61, 0.03)
Right Diaphragm thickness	Rest	0.12 ± 0.04	0.12 ± 0.03	0.12 ± 0.05	0.300	0.16	−0.01 (−0.03, 0.02)
	Contraction	0.23 ± 0.13	0.22 ± 0.11	0.24 ± 0.15	0.258	0.20	−0.03 (−0.11, 0.05)
Left Diaphragm thickness	Rest	0.16 ± 0.21	0.18 ± 0.25	0.13 ± 0.05	0.203	0.25	0.05 (−0.06, 0.15)
	Contraction	0.24 ± 0.10	0.25 ± 0.10	0.23 ± 0.09	0.183	0.28	0.03 (−0.03, 0.08)
Diaphragmatic Excursion	Rest	16.41 ± 1.62	16.58 ± 1.70	16.25 ± 1.54	0.254	0.20	0.33 (−0.66, 1.31)
	Contraction	12.52 ± 1.84	12.54 ± 2.04	12.49 ± 1.64	0.465	0.03	0.05 (−1.08, 1.17)
Diaphragmatic Excursion	Difference	3.90 ± 1.24	4.04 ± 1.30	3.76 ± 1.17	0.231	0.22	0.28 (−0.48, 1.03)

Abbreviations: CG: control group, DG: dysmenorrhea group.

**Table 2:** Respiratory measurements of the total sample and differences between groups.

Respiratory Measurements	Total Sample (n = 44)	CF (n = 22)	DG (n = 22)	*p*-Value	Cohen’s d	Mean Difference (95% CI)
MIP	78.91 ± 17.77	78.64 ± 16.49	79.18 ± 19.10	0.460	0.03	−0.55 (−11.40, 10.31)
MEP	85.32 ± 20.55	87.09 ± 21.81	83.55 ± 19.28	0.571	0.17	3.55 (−8.98, 16.07)
FVC	95.73 ± 11.27	94.27 ± 11.69	97.18 ± 10.85	0.199	0.26	−2.91 (−9.77, 3.95)
FEV1	95.47 ± 10.33	94.91 ± 9.44	96.03 ± 11.21	0.361	0.11	−1.12 (−7.43, 5.18)
FVC_FEV1	84.93 ± 7.01	85.91 ± 6.15	83.95 ± 7.86	0.182	0.28	1.95 (−2.35, 6.26)

Abbreviations: MIP: maximum inspiratory pressure; MEP: maximum expiratory Pressure; FVC: forced vital capacity; FEV1: forced expiratory volume in the first second; FVC_FEV1: ratio of forced vital capacity to forced expiratory volume in the first second); CG: control group, DG: dysmenorrhea group.

## Data Availability

The datasets supporting the findings of this study can be requested from the first author.
